# Hepatitis C Virus: An Overview of Its Chronic Impact on Liver Function, Metabolic Dysregulation, Inflammatory–Oxidative Pathogenesis and Epigenetic Memory

**DOI:** 10.3390/ijms27083559

**Published:** 2026-04-16

**Authors:** Joana Ferreira, João Caldeira, Manuel Bicho, Paula Faustino, Fátima Serejo

**Affiliations:** 1 Institute for Scientific Research Bento Rocha Cabral, 1250-047 Lisbon, Portugal; manuelbicho@medicina.ulisboa.pt; 2 Genetics Laboratory, Lisbon Medical School, University of Lisbon, 1600-190 Lisbon, Portugal; joaopedrovc01@gmail.com; 3 Environmental Health Institute (ISAMB), Lisbon Medical School, University of Lisbon, Associated Laboratory TERRA, Higher Institute of Agronomy, 1649-028 Lisbon, Portugal; paula.faustino@insa.min-saude.pt (P.F.); fatimaserejo53@gmail.com (F.S.); 4 Human Genetics Department, National Institute of Health Dr. Ricardo Jorge, 1649-016 Lisbon, Portugal; 5 Gastroenterology and Hepatology Department, Hospital de Santa Maria, 1649-028 Lisbon, Portugal

**Keywords:** chronic hepatitis C, metabolic dysregulation, inflammatory–oxidative pathogenesis, liver damage

## Abstract

Hepatitis C virus (HCV) infection is a global health concern, chronically affecting over 71 million people. It primarily targets the liver but also causes systemic complications through inflammation, oxidative stress, and metabolic dysregulation. HCV is a highly variable RNA virus with six major genotypes that are mainly transmitted via blood. Often asymptomatic, the infection progresses silently to chronic hepatitis C (CHC), which can lead to fibrosis, cirrhosis, and hepatocellular carcinoma (HCC). Direct-acting antivirals (DAAs) have revolutionized treatment, achieving cure rates above 95%, improving liver function, reversing fibrosis, and normalizing metabolism. HCV disrupts iron metabolism by suppressing hepcidin, causing iron overload and oxidative stress. It also alters lipid metabolism, inducing steatosis, and affects glucose metabolism, contributing to insulin resistance and type 2 diabetes. DAAs improve these metabolic outcomes. HCV promotes oxidative stress via viral proteins, damaging liver cells and DNA and triggering inflammation and fibrogenesis. Even post-cure, oxidative stress and iron overload may continue to drive disease progression. Genetic and epigenetic factors influence fibrosis progression and HCC risk. Despite a sustained virologic response (SVR), patients with advanced liver damage remain at risk for HCC and metabolic diseases, highlighting the need for continued monitoring and personalized post-treatment care.

## 1. Introduction

Hepatitis C virus (HCV) infection remains a major global health concern, with over 50 million people chronically infected worldwide, according to the World Health Organization (WHO). The virus is a leading cause of chronic liver disease, liver cirrhosis, and hepatocellular carcinoma (HCC) and significantly contributes to the global burden of liver-related mortality [1,2]. Although the primary organ affected is the liver, HCV is increasingly recognized as a systemic virus capable of inducing a wide spectrum of extrahepatic manifestations, including insulin resistance, iron overload, dyslipidemia, and chronic systemic inflammation. These complications result from complex interactions between viral proteins, host immune responses, and cellular stress pathways, with oxidative stress playing a central and unifying role [3].

The development of direct-acting antivirals (DAAs), which specifically target non-structural viral proteins essential for HCV replication, had revolutionized the treatment of chronic hepatitis C (CHC) with cure rates exceeding 95%, minimal side effects, and short treatment durations (8–12 weeks) [4,5].

The present review aims to provide a comprehensive synthesis of the current knowledge regarding the interplay between HCV infection and liver and metabolic pathology. Emphasis is placed on the role of HCV-induced chronicity in fibrogenesis, inflammation, and steatosis, as well as in metabolic pathways such as those of lipid, iron, and glucose metabolisms; inflammatory and immune responses; and oxidative stress. It also focuses on the emerging data on how DAA therapy alters these pathways and how it affects liver disease. It ends with recent evidence about the importance of the host genetic background in liver disease progression and hepatocellular carcinoma risk after DAA therapy and the future impact on personalized medicine to account for better surveillance and prognosis of hepatitis C patients.

By understanding the mechanisms described in these studies and their response to treatment, we can improve the clinical management of patients following DAA therapy, enhance long-term outcomes, and inform future research into adjunctive strategies aimed at restoring metabolic and oxidative balance.

## 2. Hepatitis C Virus

### 2.1. Hepatitis C Virus Structure

HCV was identified in 1989 in the USA by immunoscreening serum from a patient with post-transfusion non-A and non-B hepatitis (NANB) and is the leading cause of CHC, liver cirrhosis, and hepatocellular carcinoma worldwide [6]. It is classified within the genus Hepacivirus of the family Flaviviridae, which also includes flaviviruses, pestiviruses, and hepatitis G viruses (types A, B, and C) [7].

It contains a single-stranded ribonucleic acid (RNA) with approximately 10,000 nucleotides and is encapsulated by an outer lipid envelope. The diameter of the virus is 50–60 nm [8]. The HCV genome is enclosed in an icosahedral capsid surrounded by a double lipid layer on which two different glycoproteins are anchored. The genome contains three distinct regions: a small 5′ non-coding area containing two domains, a stem-loop structure, and an internal ribosome entry site responsible for ribosome binding and polyprotein translation. A single open reading frame (ORF) of more than 9000 nucleotides is translated into a precursor polyprotein, which is subsequently cleaved to generate both structural (the capsid protein and the two envelope glycoproteins E1 and E2) and non-structural proteins (p7, NS2, NS3, NS4A, NS4B, NS5A, and NS5B) by both host and viral proteases. Among these, the viral NS3/4A serine protease plays a critical role in cleaving the non-structural region of the polyprotein, thereby enabling the formation of the viral replication complex [9,10]. In addition to its role in viral maturation, NS3/4A also contributes to immune evasion by disrupting host innate immune signaling pathways, making it a key determinant of viral persistence [11]. Consequently, NS3/4A has been a major target for direct-acting antivirals, with protease inhibitors forming an essential component of several therapeutic regimens. NS5A is another essential non-structural protein targeted by current DAA combinations such as sofosbuvir/velpatasvir and glecaprevir/pibrentasvir. Although NS5A lacks known enzymatic activity, it is a multifunctional phosphoprotein involved in viral RNA replication, the assembly of virions, and the modulation of host cell pathways [12,13]. Its precise mechanisms of action remain incompletely understood, which has complicated drug development efforts. NS5A inhibitors are highly potent but are associated with a low barrier to resistance, as single amino acid substitutions can significantly reduce drug susceptibility [14]. Despite these challenges, NS5A inhibitors remain a cornerstone of highly effective combination therapies due to their ability to disrupt multiple stages of the viral life cycle. The functions of the non-structural proteins have been largely characterized, although some—particularly NS4B and NS5A—remain incompletely understood. The genome also contains a short non-coding region (3′UTR) that is divided into three regions: a variable sequence of about 40 bases, a poly-UC part, and a highly conserved region of 98 bases [15,16,17,18].

In the plasma of an infected patient, there are approximately 105–107 viral particles/mL of blood, and in 15% of patients, the number can even rise to 109 particles/mL. The number of viruses in other body fluids and tissues is much lower [8].

### 2.2. Hepatitis C Virus Genotypes

HCV has a half-life of only a few hours, and HCV infection is a highly dynamic process and accounts for a release of about 10^12^ viral particles per day [19]. This high replicative activity and the lack of a proofreading function underline the high genetic variability of HCV. HCV is a highly heterogeneous virus and can be grouped into genotypes and subtypes. It has at least six genotypes, designated 1 to 6, and more than 50 subtypes, defined by letters (e.g., 1a, 1b, 2a, and 2b), and its only known host is humans. The nucleotide sequences of the individual virus genotypes differ by approximately 31–34% and by 20–23% in subtypes. Due to high genomic variability and frequent mutations, the HCV population in an infected patient is heterogeneous and helps the virus survive the mechanisms of the host immune system [8,15]. Over 40,000 HCV sequences have been identified [18].

The genotypes with the most significant global distribution are genotypes 1, 2, and 3. In contrast, genotype 4 is more restricted in Middle Eastern countries and Africa, particularly in countries like Saudi Arabia, Bahrain, Jordan, Egypt, and Ethiopia. Genotypes 5 and 6 have been found in South and East Africa and Southeast Asia. In Europe, the predominant genotype is genotype 1, with 64.4%, followed by genotype 3, with 25.5%, then genotype 2, with 5.5%, and finally genotype 4, with 3.7%. The other genotypes were found in insignificant percentages [20].

The most dominant genotypes in Portugal are genotypes 1 and 3 [21], with a prevalence of 62.6% and 18.3%, respectively [16]. Within genotype 1, the most frequent subtypes were 1a, with 75.5%, and 1b, with 24.5% [22].

Genotype 1 is more prevalent in individuals with a history of blood transfusions or who have undergone surgical procedures. Genotype 2 is mainly associated with hospital infections and dental treatments. Genotype 3 is often found in groups of intravenous drug abusers. Finally, genotype 4 is transmitted mainly through unsafe sexual practices and intravenous drug use. Infection with genotype 3 is associated with more rapid progression of liver fibrosis, a higher degree of steatosis, and a higher incidence of cirrhosis and hepatocellular carcinoma. Spontaneous clearance is observed more frequently with HCV genotype 1 infection. Still, when individuals with this genotype remain infected with the virus, there is more aggressive disease progression than with the other genotypes. Genotypes 1 and 4 are associated with low response rates and a longer duration of treatment with interferon–ribavirin combination therapy compared to genotypes 2 and 3 [23].

### 2.3. Hepatitis C Virus Life Cycle

The HCV life cycle begins with attachment to a specific receptor. Several molecules that play a role in the receptor complex have already been suggested, such as CD81, a protein found on the surface of many cell types, including hepatocytes and the low-density lipoprotein receptor (LDLR); the scavenger receptor class I type B (SR-BI); claudin-1 (CLDN1); glycosaminoglycans (GAGs); and Niemann–Pick C1-Like 1 (NPC1L1) [18,24,25,26].

Viral particles circulate in serum as triglyceride-rich particles. Consequently, lipoproteins and their receptors play an essential role in virus internalization and the initiation of infection. SR-BI is a receptor with multiple ligands for several classes of lipoproteins (high density lipoprotein (HDL), low-density lipoprotein (LDL), and very low-density lipoprotein (VLDL)) and modified lipoproteins (oxidized LDL (LDLox) and acetylated LDL). The essential function of SR-BI is the selective uptake of cholesterol esters (CEs) from HDL. The cholesterol flux mediated by SR-BI is essential in reverse cholesterol transport and atherogenesis [24].

After binding to various host cell components, the HCV particle is internalized by clathrin-mediated endocytosis. Various serum components can modulate HCV entry. The lipid composition of hepatocyte and virus envelope glycoproteins plays a crucial role in HCV recognition and virus entry into cells [24]. Indeed, liver-secreted serum amyloid apoprotein (SAA), LDLox, and lipoprotein lipase (LPL) appear to inhibit HCV entry. In contrast, HDL, the primary ligand of SR-BI, facilitates HCV entry into hepatocytes (a 3-fold increase in HCV entry efficiency was observed in the presence of HDL). Still, there is no direct interaction between HDL and viral particles. Furthermore, increased HDL expression upon HCV entry reduces the sensitivity of the virus to neutralizing antibodies [25,26]. Apolipoproteins B, E, and C1 (Apo B, Apo E, and Apo C1), constituents of VLDL, are also efficient regulators of HCV infectivity, promoting the fusion of the viral membrane with the host cell membrane [24].

When HCV enters the cell, the virus releases the HCV-RNA viral genome from the capsid, which is used for translating proteins and for replication in the cytoplasm [4]. Replication and post-transduction processes occur in a membranous web (Mw) of non-structural proteins and host cell proteins located next to the perinuclear membranes. Translated viral proteins use the genomic RNA to produce negative-strand RNA, which is then used as a template to synthesize new genomic RNA strands [16]. Capsid encapsulation (assembly) appears to occur in the endoplasmic reticulum (ER), and maturation and envelope formation in the Golgi apparatus [18] occur before the newly produced virions are released into the pericellular space by exocytosis [15,19,27].

## 3. Hepatitis C Virus Infection and Outcome

### 3.1. Hepatitis C Virus: From Infection to Chronicity

The WHO estimates that about 50 million people have CHC virus infection with about 1.0 million new infections occurring per year. Rather than representing a single disease entity, CHC constitutes a clinical and pathological syndrome arising from multiple etiologies and is defined by variable degrees of hepatocellular necrosis and inflammation [1,28].

Globally, CHC remains a major contributor to liver transplantation requirements and liver-related mortality [16]. Approximately 60–85% of individuals exposed to HCV progress to chronic infection, with an estimated 15–30% developing liver cirrhosis or HCC within two decades. Among those with established cirrhosis, the annual incidence of HCC ranges from 1% to 3%, whereas the risk of hepatic decompensation is estimated at 3–6% per year. Once decompensation occurs, mortality within the subsequent 12 months rises to 15–20% [1,2]. HCC represents one of the principal causes of liver-related death and ranks as the third leading cause of cancer mortality worldwide [29].

Hepatic alterations in CHC generally follow a pattern of slowly advancing fibrosis, with the transition from stage 0 (no fibrosis) to stage 4 (cirrhosis) occurring at an average rate of 0.10–0.15 fibrosis units per decade. Fibrogenesis represents a nonspecific and frequently disproportionate reaction to hepatic injury [30].

The clinical course and outcomes of CHC—including the extent of fibrosis and related complications—are modulated by host characteristics (such as age, sex, obesity, hepatic steatosis, and genetic background), viral determinants (including genotype and viral load), and environmental influences such as alcohol intake [29,31].

Age is an essential factor to consider in the progression of fibrosis stages. It has been shown in several studies that the occurrence of HCV infection at older ages (>40 years) leads to more rapid progression of fibrosis. The reasons for age-related differences in fibrosis progression are unclear. Still, it is believed that these differences may be influenced by changes in physiological or immunological mechanisms [31,32].

Concerning gender, several studies have shown that females have a higher rate of spontaneous resolution (45%) of acute HCV infection than males. Gender will also influence the progression of advanced liver disease through hormonal differences. For example, higher serum testosterone levels are associated with more severe stages of liver fibrosis, and studies have proposed that estrogen plays a protective role in females. In addition, results obtained in vitro suggest that estrogen modifies hepatocellular cancer, with a higher risk for males. Sex-based hormonal differences significantly influence extracellular matrix (ECM) synthesis and the progression of hepatic fibrosis. Premenopausal women, who have higher circulating estrogen levels, exhibit estrogen-mediated inhibition of hepatic stellate cell (HSC) activation, lower deposition of collagen types I and III, enhanced expression of matrix metalloproteinases (MMPs) relative to their inhibitors (TIMPs), and reduced TGF β/Smad3 profibrotic signaling. This results in markedly less ECM accumulation compared to males, who generally have lower MMP/TIMP ratios, more robust HSC activation, and greater collagen deposition in response to liver injury. Consequently, men tend to develop more severe and faster-progressing hepatic fibrosis than women, especially before menopause, a period when estrogen’s anti-fibrotic effects are present [31,32,33].

Obesity is an independent risk factor for fibrosis progression to high stages. Studies have shown that individuals with a body mass index (BMI) greater than 25 kg/m^2^ rapidly progressed in the fibrosis stages. Obesity has also been found to be a risk factor because it contributes to non-response to antiviral therapies [31,32].

Steatosis is the abnormal accumulation of fat in liver cells. It is a disease with some prevalence in the general population and is two to three times more common in individuals with CHC. The cause of steatosis in individuals with hepatitis C is multifactorial, ranging from metabolic disorders to obesity. Steatosis promotes the development of fibrosis and accelerates its progression [31].

Regarding viral factors, there is little evidence that the HCV viral load and genotype affect the evolution of CHC. Most studies have observed no correlation between virus RNA levels and histological findings. On the other hand, one study suggested that HCV genotype 3 may be associated with an acceleration of fibrosis progression [32].

One of the most relevant environmental factors for the progression of liver disease in patients with HCC is alcohol consumption. Consumption of this additive is known to affect the outcome of HCV infection adversely and is associated with more rapid progression of liver fibrosis [29]. Alcohol consumption profoundly accelerates the progression of liver disease via multiple interconnected mechanisms. Chronic ethanol intake leads to accumulation of toxic metabolites such as acetaldehyde and reactive oxygen species, resulting in hepatocyte injury, oxidative stress, lipid peroxidation, and activation of pro-inflammatory cytokines. These processes stimulate hepatic stellate cells to produce excess ECM, driving fibrosis and ultimately cirrhosis. Studies from the past few years further indicate that even low-to-moderate drinking synergizes with metabolic risk factors (like obesity and diabetes), exponentially increasing fibrosis risk compared to non-drinkers [34,35].

HCV infection is recognized as a systemic condition rather than a disease confined solely to the liver. Approximately 74% of affected individuals exhibit extrahepatic manifestations of varying clinical severity [36,37,38,39,40,41,42]. These manifestations encompass a wide array of organ systems, contributing to complications such as cardiovascular disorders, renal dysfunction, diabetes mellitus, insulin resistance, chronic systemic inflammation, oxidative stress, iron accumulation, and lipid metabolism disturbances. Although hepatic pathology remains the predominant cause of death among patients with HCV infection, mortality related to extrahepatic comorbidities also occurs at a significantly elevated rate within this population [3].

### 3.2. Effects of Hepatitis C Virus Chronicity on Liver: Fibrosis and Liver Disease Markers

Once HCV successfully circumvents immune surveillance, it establishes infection within hepatocytes, leading to enhanced oxidative stress and subsequent recruitment of inflammatory cell populations. This inflammatory cascade promotes the activation of hepatic myofibroblasts, resulting in excessive collagen deposition. Moreover, HCV-encoded proteins stimulate hepatocytes to release profibrotic cytokines, which, in turn, exert direct fibrogenic effects on hepatic stellate cells. The combined outcome of these processes is the progressive development of liver fibrosis, which is characterized by persistent hepatocellular injury and the accumulation of extracellular matrix components. This accumulation will distort the liver architecture, creating scarring that subsequently leads to the development of hepatocyte nodules, thus defining liver cirrhosis. In addition, cirrhosis produces hepatocellular dysfunction and increased intrahepatic resistance to blood flow, thus resulting in liver failure and portal hypertension [43].

Historically, hepatic fibrosis was considered a passive and irreversible process due to the collapse of the liver parenchyma and its replacement by collagen-rich tissue. However, it is known that fibrogenesis in the liver is an active healing process of hepatic lesions and may be reversible [43,44].

The pathophysiology of fibrosis involves chronic liver damage and requires the interaction of several types of liver cells. It involves hepatocyte damage and cell death, Kupffer cell (KC) and HSC activation, and chronic inflammation. HSCs activated by fibrogenic cytokines (e.g., transforming growth factor beta (TGF-β) and tumor necrosis factor alpha (TNF-α)) have been identified as the significant collagen-producing cells in the injured liver [45]. Hepatic fibrosis is, therefore, characterize by excessive accumulation of extracellular matrix proteins in the liver parenchyma, such as collagen, laminin, elastin, and fibronectin [44,46]. Liver damage causes HSCs to become more active, which triggers an increase in extracellular matrix synthesis [44]. Not only does it increase the average amounts of collagen fiber deposits (predominantly collagen I and III) in the extracellular spaces of liver cells, but it also causes changes in hepatic blood flow with a reduction in oxygen and nutrient delivery to liver cells. This stresses the cells, causing them to change their internal structure (cytoskeleton), making them stiffer and more rigid, and contributing to the worsening of liver fibrosis [44,47]. First, HSCs become directly fibrogenic, increasing the synthesis and deposition of the extracellular matrix. It follows the proliferation of fibrogenic cells and, consequently, a worsening of liver function, as exemplified by the compromised ability to eliminate toxins and synthesize the critical proteins, lipids, and carbohydrates necessary for maintaining bodily functions [47]. The final consequences are liver dysfunction, portal hypertension, and an increased risk of HCC [48].

The effects of HCV elimination with DAAs on the natural history of liver disease are not clear in the literature because most studies regarding these effects include a small number of patients and have a short follow-up duration.

A 2020 investigation analyzing 40 paired liver biopsy samples from patients with CHC before and after DAA therapy demonstrated a marked reduction in hepatic inflammation and fibrosis following HCV eradication [49]. In the same year, a large-scale prospective cohort study including over 2000 individuals with liver fibrosis, monitored for at least one-year post-treatment, reported that viral clearance achieved through DAA therapy in CHC was linked to cirrhosis reversal and regression of hepatic fibrosis in approximately 50% of cases [50]. More recently, data published in 2024 indicated that successful elimination of HCV via DAAs leads to decreased liver stiffness and attenuation of fibrosis among CHC patients receiving antiviral therapy [51].

Hepatic injury resulting from HCV infection induces structural alterations within the liver, manifesting not only as progressive stages of fibrosis but also as elevations in circulating noninvasive biomarkers reflecting hepatocellular injury, necroinflammatory activity, and fibrotic remodeling [52]. These biomarkers can aid in diagnosis, assessment of disease severity, monitoring of the therapeutic response, and evaluation of liver disease prognosis. Based on those results, the measurement of aspartate alanine aminotransferase (ALT) and aspartate aminotransferase (AST) has a prominent role.

ALT and AST are enzymes present in hepatocytes that play a role in amino acid metabolism and facilitate entry into the citric acid cycle. They are widely used as diagnostic biomarkers of liver injury, as hepatocellular damage leads to their release into the bloodstream [31,52]. However, while ALT is relatively liver-specific, AST is also present in extrahepatic tissues such as cardiac and skeletal muscle, and its elevation may reflect injury beyond the liver. ALT is considered a marker of necroinflammation, and increased hepatic inflammation is associated with enhanced fibrogenesis [31,53,54,55,56]. Several studies have reported higher AST and ALT levels in advanced stages of liver fibrosis. Moreover, elevated ALT levels are associated with faster progression of fibrosis, highlighting the importance of monitoring this enzyme [56].

Gamma-glutamyl-transpeptidase (γGT) is a cholestatic enzyme as it is primarily associated with the bile ducts and the epithelial cells lining them. It is predominant in the liver, and it is responsible for the catabolism of extracellular glutathione (GSH) and other γ-glutamyl compounds. γGT expression increases during the process of hepatic fibrogenesis, reflecting the existence of lesions in the biliary ducts in patients with severe fibrosis. Moreover, its elevation may account, in part, for the adaptation and over-compensation of oxidative stress, which is more evident in higher fibrosis stages [48,57,58,59].

Elevated γGT expression is not only a marker of hepatocellular damage but also a participant in oxidative stress pathways that contribute to liver fibrogenesis. Through glutathione catabolism, it produces cysteinyl-glycine, a thiol that can act as a pro-oxidant under certain conditions, especially in the presence of metal ions. This promotes the oxidation of LDL, forming oxidized LDL (oxLDL), which is known to activate inflammatory responses and hepatic stellate cells, key drivers of fibrogenesis [60,61].

### 3.3. Hepatitis C Virus Elimination

The prevailing therapeutic standard for HCV infection involves the administration of direct-acting antivirals (DAAs). These compounds specifically disrupt the viral replication process by inhibiting the key nonstructural proteins required for genome replication. In adult populations, recommended treatment courses generally extend over 8 to 12 weeks. Evaluation of treatment efficacy should include quantification of HCV-RNA and assessment of serum aminotransferase levels at 12 or 24 weeks following completion of DAA therapy. The absence of detectable HCV-RNA denotes a sustained virologic response (SVR), representing definitive viral eradication. Achievement of the SVR is typically correlated with normalization of hepatic enzyme concentrations, attenuation or reversal of hepatic necroinflammatory activity and fibrosis, and overall improvement in liver function. DAAs have revolutionized the treatment of HCV by offering highly effective, well-tolerated, and short-duration therapies. One of the major advancements in this field is the development of pangenotypic DAAs, which are effective across all HCV genotypes. These agents, such as sofosbuvir/velpatasvir and glecaprevir/pibrentasvir, target essential viral proteins and have shown cure rates exceeding 95% regardless of genotype, making them a cornerstone in global HCV elimination strategies [4,5,62,63].

## 4. Effects of Hepatitis C Virus Chronicity on Metabolism

### 4.1. Acute Inflammatory Response: Role of Haptoglobin

Haptoglobin (Hp) is an acute-phase glycoprotein with the capacity to bind free hemoglobin in the circulation, thereby preventing oxidative damage. In addition to its hemoglobin-scavenging function, Hp exhibits notable antioxidant and antibacterial properties. Under inflammatory conditions, its plasma concentration increases both at the vascular (local) and extravascular (systemic) levels [64].

In humans, Hp gene expression is primarily regulated by interleukin-6 (IL-6), which activates transcription through three IL-6–responsive elements—designated regions A (-157), B (-111), and C (-61)—located within the Hp gene promoter. These regulatory elements serve as binding sites for the cytokines and hormones released during the acute-phase response, thereby stimulating Hp gene transcription and enhancing protein synthesis [64].

During the early phase of an acute inflammatory response, Hp actively participates by binding to the macrophage-1 antigen (Mac-1) receptor on immune cells. This interaction helps regulate the initial recruitment of neutrophils to the site of inflammation. By binding on Mac-1, Hp dampens neutrophil activity, reducing excessive inflammatory damage. As the response progresses, Hp further contributes to immune regulation by promoting neutrophil apoptosis, which is essential for resolving inflammation. During tissue injury, Hp binds the free hemoglobin released, forming Hb–Hp complexes that are cleared via the CD163 receptor on monocyte-derived macrophages. This uptake induces heme oxygenase-1 (HO-1) expression, promoting anti-inflammatory signaling and contributing to the polarization of macrophages towards an M2-like reparative phenotype, which is characterized by interleukine-10 (IL-10) and TGF-β production and enhanced tissue remodeling capacity [65,66,67]. This mechanism is essential for limiting oxidative damage and resolving inflammation, particularly in hemolytic and metabolic stress contexts [68]. As tissue healing advances, haptoglobin expression increases, further supporting the repair process by inhibiting gelatin matrix deposition and limiting fibroblast migration—two key steps in controlling excessive scarring and promoting organized wound healing [64].

It is known that CHC patients also have signs of mild hemolysis, as indicated by significantly higher heme levels and lower haptoglobin expression [64].

**Figure 1:**
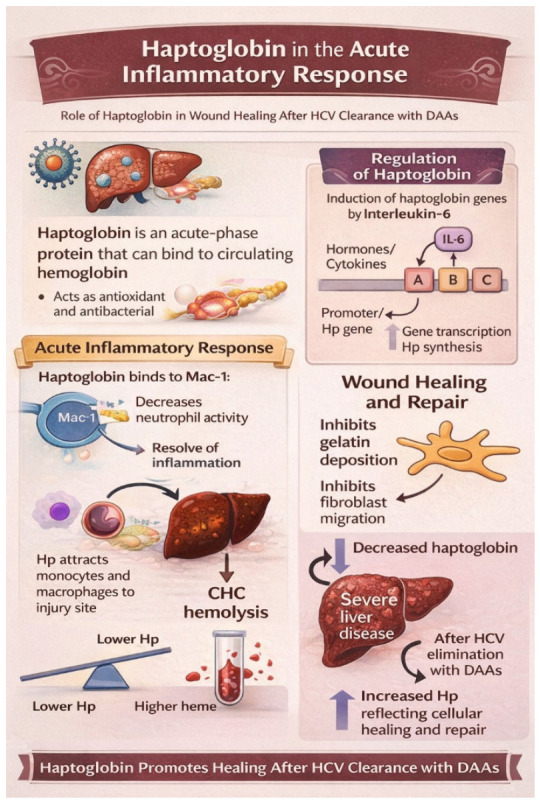
Schematic overview of the biological functions and regulatory mechanisms of haptoglobin. Hp: haptoglobin; IL-6: interleukin-6; Mac-1: macrophage-1 antigen; CHC hemolysis: chronic hepatitis C hemolysis.

Serum levels of this acute phase protein were shown to be lower in individuals with more severe liver disease. The decreased Hp values in higher fibrosis may be explained by the increased levels of the hepatocyte growth factor, which stimulates a decline in haptoglobin synthesis [61,69,70]. After HCV elimination with DAAs, haptoglobin expression increases, probably reflecting a process of cellular healing and repair [64,71] (Figure 1).

### 4.2. Immune Response: Role of Cytokines

The hepatic inflammatory response induced by HCV involves multiple mechanisms. Central to this process are host immune regulatory pathways mediated by cytokines that orchestrate antiviral defense, as well as viral proteins that interact with both innate and adaptive immune cells of the host [31]. In CHC, these immune responses are progressively dysregulated over time, contributing to disease advancement. Persistent viral infection and inflammation promote hepatocyte injury and establish a proinflammatory and profibrotic cytokine milieu. This environment stimulates hepatic stellate cells, myofibroblasts, and fibroblasts, leading to extracellular matrix accumulation and the development of hepatic fibrosis [72].

**Figure 2:**
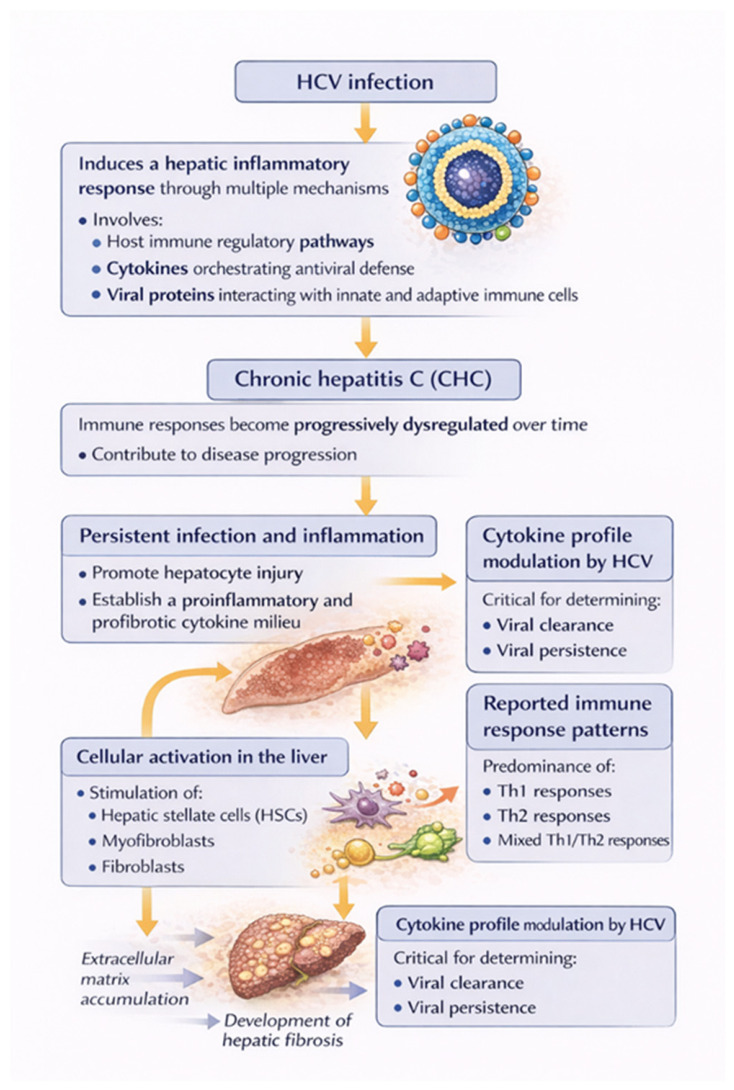
Immune response in chronic hepatitis C and its role on the development of liver fibrosis. HCV: hepatitis C virus; CHC: chronic hepatitis C; HSC: hepatic stellated cell.

HCV infection modulates cytokine profiles, and elucidating these alterations is critical for understanding the determinants of viral clearance versus persistence. However, investigations have reported divergent findings regarding the predominance of Th1, Th2, or mixed cytokine responses. Such discrepancies may stem from viral factors, including genotype and virulence, as well as host-specific variables, such as disease duration, genetic background, immune status, and the extent of hepatic fibrosis and inflammation [73,74,75] (Figure 2).

The antiviral activity of TNF-α is primarily linked to its capacity to promote lymphocyte proliferation and to enhance the targeted cytotoxic T lymphocyte (CTL) response against HCV within the hepatic environment [73]. In contrast, a Th2-skewed immune profile is predominantly humoral and can suppress antiviral effector mechanisms, thereby contributing to viral persistence and disease progression. Th2 cytokines typically inhibit Th1-driven responses following acute viral infection while also playing a protective role by limiting Th1-mediated tissue injury [73]. Lucey et al. proposed that a shift from Th1 to Th2 cytokine dominance may be involved in the pathogenesis and chronicity of viral infections [69]. Several studies have further observed elevated Th2 cytokine levels in CHC patients, which may reflect either a systemic immune response or increased local hepatic production with subsequent secretion into the circulation [76,77,78,79].

In the context of hepatitis C immunopathogenesis, the balance between pro-inflammatory Th1 and anti-inflammatory Th2 cytokines is critical, influencing the course of chronic liver disease and potentially serving as a marker for disease progression and response to antiviral therapy. Despite robust Th1 responses in HCV-infected individuals, the virus often persists due to its high mutation rate. Under these circumstances, the host immune system maintains a Th1-skewed response that is characterized by continued release of interleukine-2 (IL-2), interferon gamma (IFN-γ), and TNF-α, which enhances antiviral defense but simultaneously drives chronic inflammation, hepatocyte necrosis, and liver injury [73].

TNF-α exerts a pivotal influence in orchestrating hepatic inflammatory responses. As a key pro-inflammatory cytokine and potent immunoregulatory mediator, TNF-α is released during acute-phase inflammatory reactions. A study from 2023 demonstrated elevated concentrations of this cytokine in patients with chronic hepatitis C [80]. Similarly, Crespo J et al. identified increased intrahepatic expression of TNF-α mRNA and other cytokine transcripts, with expression levels showing a positive correlation with the degree of hepatic fibrosis [81].

Evidence from multiple investigations indicates that patients with HCV infection and hepatocellular carcinoma exhibit significantly increased TNF-α levels, underscoring its crucial involvement in both hepatocarcinogenesis and HCV-related liver pathology. Elevated TNF-α concentrations have been linked to enhanced inflammatory activity and greater disease severity in CHC. Additionally, TNF-α has been shown to participate in the activation of hepatic stellate cells and to induce phenotypic alterations in activated myofibroblasts, promoting ECM deposition and contributing to the processes of fibrogenesis and fibrosis progression [31].

TGF-β is a cytokine secreted by HSCs, fibroblasts, KCs, and M2 macrophages, functioning in the modulation of cellular growth, differentiation, and proliferation [82]. It serves as a pivotal mediator in chronic liver disorders and plays a fundamental role in fibrogenic mechanisms. Elevated TGF-β levels, stimulated by hepatic injury and platelet activation, drive the activation of HSCs and fibroblasts, leading to the emergence of myofibroblasts and the subsequent deposition of ECM components [83]. This cytokine exerts multiple biological actions, including profibrotic, anti-inflammatory, and immunosuppressive effects. The equilibrium between these effects is essential for maintaining tissue homeostasis. Aberrant TGF-β expression contributes to excessive fibrogenesis and fibrosis progression, whereas its deficiency may also result in pathological manifestations [46]. In hepatic fibrogenesis, TGF-β acts as a major inducer of ECM accumulation and tissue remodeling [26].

IL-10 is a multifunctional immunoregulatory cytokine predominantly produced by monocytes, macrophages, and T lymphocytes. As a Th2-type anti-inflammatory cytokine, IL-10 plays a key role in modulating immune activity through its suppressive and anti-fibrotic properties. It regulates the Th1/Th2 balance by downregulating Th1 responses and inhibiting pro-inflammatory cytokine production [84,85]. IL-10 is considered a critical mediator of the host immune defense against HCV infection [84,85,86]. Evidence suggests that IL-10 is involved in multiple stages of chronic liver disease, spanning from early hepatic injury and inflammation to fibrosis, cirrhosis, and hepatocellular carcinoma [83]. Both deficiency and overproduction of IL-10 are associated with pathological consequences: reduced IL-10 levels enhance inflammatory activity and may facilitate spontaneous HCV clearance, whereas excessive IL-10 expression can impair immune defense, promote viral persistence, and increase reinfection susceptibility [85].

Experimental findings have associated IL-10 with HCV persistence and the transition to chronic infection, primarily through the suppression of HCV-specific effector CD4^+^ and CD8^+^ T lymphocytes during early infection stages [87,88,89]. Despite limited evidence, IL-10 appears to exert a protective role against fibrosis. An inverse relationship between IL-10 levels and fibrosis stages has been documented in CHC patients, and IL-10 agonist therapy has been shown to attenuate fibrotic progression in HCV infection [31,79]. Elevated IL-10 concentrations have been reported in individuals with chronic hepatitis B and C compared to healthy controls. Moreover, higher IL-10 expression was observed in patients with mild hepatitis C relative to those with advanced disease, suggesting an association between reduced IL-10 levels and CHC progression. IL-10 mitigates the expression of pro-inflammatory cytokines (IL-2, IFN-γ, and TNF-α) in T cells and downregulates collagen I synthesis, thereby exerting its antifibrotic effects [90].

### 4.3. Lipid Metabolism

Lipids are a large group of macromolecules that play a relevant role in cell physiology and diseases. Within a cell, they have three essential functions: being a reservoir for neutral lipids, promoting the homeostasis of membranes and proteins, and participating in inter-organelle crosstalk [91].

HCV infection promotes the intracellular accumulation of cytosolic lipid droplets (cLDs) within hepatocytes and induces membrane remodeling through alterations in lipid composition. These modifications create structural platforms that facilitate efficient viral replication and morphogenesis. Furthermore, HCV infection has been shown to enhance the synthesis of long-chain fatty acyl species in triglycerides (TGs) and phosphatidylcholines (PCs), while increased concentrations of polyunsaturated fatty acids (PUFAs) have been observed in HCV-infected hepatocyte cultures [91].

**Figure 3:**
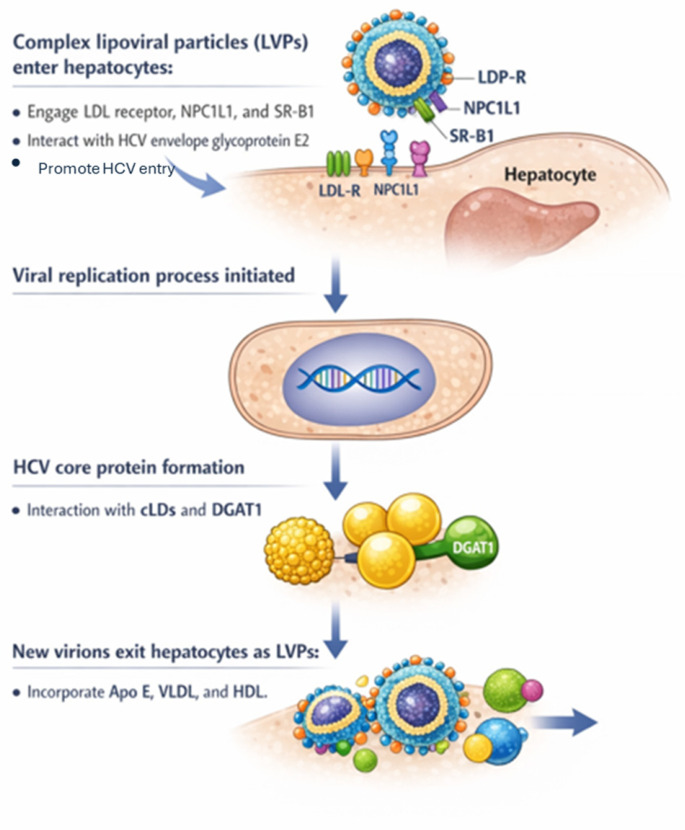
Role of lipid metabolism in HCV replication. HCV: hepatitis C virus; LVPs: lipoviral particles; LDL-R: low-density lipoprotein receptor; NPC1L1: Niemann–Pick C1-Like 1; SR-B1: scavenger receptor class B type 1; cLDs: cytosolic lipid droplets; DGAT1: diacylglycerol O-acetyltransferase 1; Apo E: apolipoprotein E; LDL: low-density lipoprotein; VLDL: very low-density lipoprotein; HDL: high-density lipoprotein.

Lipids play a pivotal role in all stages of the HCV life cycle, including entry, replication, and assembly, as well as in viral circulation through the formation of complex lipoviral particles (LVPs) [92,93]. These particles invade hepatocytes by engaging multiple receptors, such as the LDL receptor, and cell surface molecules including NPC1L1 and SRB-1, which promote cholesterol uptake from lipoproteins and interact with HCV envelope glycoprotein E2 to promote HCV entry. Several apolipoproteins may influence HCV uptake: Apo C1 interacts with HCV glycoproteins to promote infection, and Apo E mediates the initial attachment between the virus and the hepatocyte. Another mechanism for HCV entry is mediated by the VLDL receptor and involves the HCV envelope glycoprotein E2 and Apo E. Once in hepatocytes, the replication process must be initiated, and the formation of the HCV core protein consists of the interaction with host cLDs and diacylglycerol O-acetyltransferase 1 (DGAT1), a host enzyme that catalyzes the covalent attachment of fatty acyl-CoA and diacylglycerol (DAG), the final step of triglyceride synthesis. HCV hijacks cLD metabolism and changes the intracellular lipid species pool, enhancing its life cycles [92]. Newly formed virions leave hepatocytes as LVPs that incorporate Apo E-containing lipoproteins, mainly VLDL and HDL [94] (Figure 3).

Much evidence points to a profound impact of HCV infection on lipid metabolism, even in the presence of other comorbidities like type 2 diabetes mellitus (T2D) [95].

It is well known that HCV-infected patients have altered lipid profiles that are due to the interaction between HCV and host cholesterol synthesis pathways [96]. HCV infection induces hypolipidemia and steatosis. Low total cholesterol (TC), LDL, near-normal TG, and HDL expression are metabolic changes associated with HCV infection that rapidly reverse after SVR with DAAs. Previous studies showed that an increase in LDL after DAAs was related to carotid intima-media thickening and cardio-cerebral events in a relatively short follow-up of 26 months [97,98]. Another one reported a pro-atherogenic lipid pattern in patients infected with HCV during DAA therapy treatment and in a short time [99,100]. There is clear evidence that HCV eradication produces a simultaneous increase in serum TC and LDL levels, creating a combination of circumstances that might aggravate the risk of atherosclerosis and cardiovascular diseases [101]. Regarding HDL, the results of the different studies are contradictory since some show increases in HDL levels, while others show decreases, and some offer no significant differences [102,103,104]. In the case of TG, contradictory results have also been reported, with minimal or absent changes [103,105] or a decrease [106,107].

A study from 2022 reported that TC and LDL showed the earliest and most pronounced increase after DAA therapy, and this result was aligned with most studies, including retrospective studies with short follow-ups or heterogeneous populations. On the other hand, triglycerides and HDL showed less abrupt and more gradual increases [99]. These results are aligned with other studies with long follow-ups [103,107,108]. Interestingly, studies that reported no differences in HDL and TG, such as those from Ichikawa et al., Cheng et al. and Jain et al., had reduced follow-ups [97,109,110].

Statin therapy significantly reduces the progression of liver disease and mortality in HCV-infected patients. A large retrospective cohort found a ~45% lower hazard of liver decompensation and death in statin users with HCV-related cirrhosis [111]. Other studies suggest that statins are linked to a lower risk of decompensation, variceal bleeding, and mortality in chronic liver disease including compensated cirrhosis and reveal lower rates of new liver disease and liver-related deaths among regular statin users [109,112,113].

Excessive accumulation of triglycerides within hepatocytes may lead to hepatic steatosis, and HCV is one of the causes. It affects approximately half of HCV-infected individuals and results from the interaction between several host and viral factors that directly interfere with lipid metabolism within the hepatocytes. Steatosis can be a relevant factor for the aggravation of liver disease in CHC, leading to fibrosis and progressing to cirrhosis and HCC [114].

**Figure 4:**
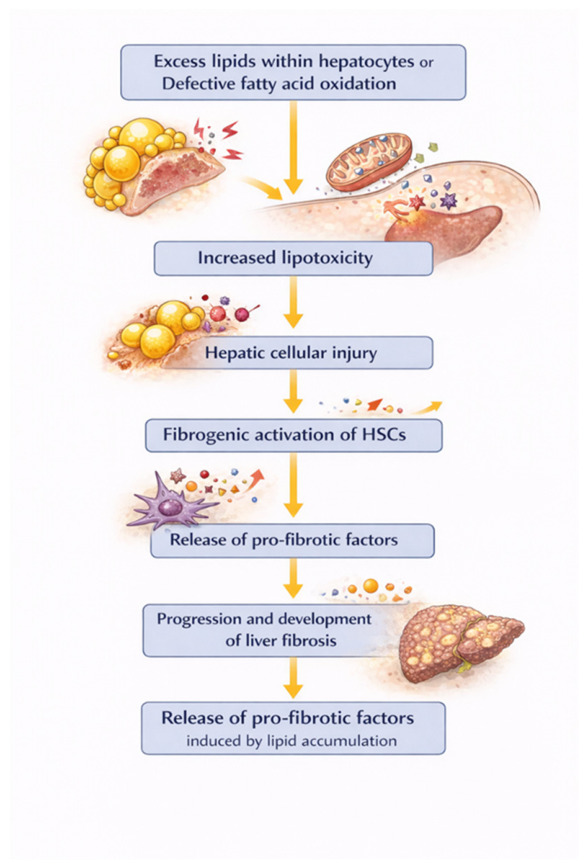
Role of lipids in promoting liver fibrosis. HSCs: hepatic stellated cells.

There is evidence that excess lipids within hepatocytes and defective fatty acid oxidation are associated with increased lipotoxicity, which directly contributes to the development of fibrosis [115]. Lipid accumulation in hepatocytes induced fibrogenic activation of HSCs, which is accelerated by specific profibrotic factors caused by lipid accumulation in hepatocytes [116] (Figure 4).

### 4.4. Iron Metabolism

Iron is a physiologically essential nutrient for humans and vital for cellular homeostasis as it functions critically in many cellular processes. It is crucial for oxygen transport, deoxyribonucleic acid (DNA) synthesis, and energy metabolism, and it is an essential cofactor for enzymes in the mitochondrial respiratory chain as well as in nitrogen fixation [117,118,119]. In mammals, its primary function is the synthesis of hemoglobin in the erythroblasts, myoglobin in the muscles, and cytochromes in the liver [119].

However, iron can be biochemically dangerous because, in excess, it will damage tissues by promoting the synthesis of toxic reactive oxygen species to cell membranes, proteins, lipids, and DNA [119,120]. Under iron overload conditions, the liver is the main receptor organ for this metal. High liver iron concentrations can result in hepatocellular lesions, fibrosis, and cirrhosis [121]. Iron deficiency is also a worrying situation, compromising all the above functions and having consequences for the whole organism [117,119,121].

Iron is obtained from diet, and two types of this metal can be found in food: hemic iron, which is mainly present in red meat, poultry, and fish, and non-hemic iron, which is mainly present in green leafy vegetables, legumes, fruits, and dairy products [117,119].

The iron absorption process takes place in the enterocytes of the intestinal duodenum. It is divided into two fundamental stages: absorption through the apical membrane and passage into the bloodstream via the basolateral membrane.

Non-hemic iron in the diet is generally in the ferric form (Fe^3+^), which the body does not assimilate well. To be absorbed by the enterocyte, it is reduced to the ferrous form (Fe^2+^) by duodenal cytochrome b (DCYTB), whose expression increases during iron deficiency or hypoxia, playing an essential role in iron absorption. The acidic pH of the apical membrane allows Fe^2+^ to enter the enterocyte through divalent metal transporter 1 (DMT1), which is responsible for the transport of iron and other metals such as cobalt, zinc, or cadmium across the apical membrane [122,123,124].

Heme iron is transported to the enterocyte by the action of a transporter protein that carries the entire Heme group, namely heme carrier protein 1 (HCP1). The expression of this protein increases in case of iron deficiency or hypoxia. Upon reaching the cytoplasm of the enterocyte, the heme group is separated by the enzyme HO-1, thus releasing Fe^2+^ [122]. Once in the cytoplasm, iron can be stored by ferritin (Ft) or be exported by ferroportin (FPN1), a protein located in the basolateral membrane and the only cellular exporter of iron. Hephaestin oxidizes Fe^2+^ to Fe^3+^, which is subsequently incorporated into transferrin (Tf) [122,123,124]. Iron bound to transferrin is transported through the bloodstream to perform various functions, mainly to enable erythropoiesis in bone marrow.

Tf is found in plasma in three form, namely apo-transferrin (apo-Tf) (when there is no iron-binding activity), monoferric transferrin (Tf-Fe^3+^) (binding to a single iron atom), and diferric transferrin (Tf-(Fe^3+^)_2_) (binding to two iron atoms), allowing the response to abrupt increases in iron absorption, thereby preventing the toxic effects of excess iron in the body [125]. Tf-Fe_2_ and Tf-Fe complexes bind to the transferrin receptors (TfRs) located on the plasma membrane of the cells, resulting in a new Fe-Tf-TfR complex. This complex invaginates to form a clathrin-coated endosome. Then, through a proton pump, the pH of the endosome drops, causing conformational changes in Tf and TfR, which promote the release of Fe^3+^. This ion is reduced to Fe^2+^ and transported to the cytoplasm by DMT1. The Apo-Tf-TfR complex, is then recycled back to the cell surface, where apo-transferrin dissociates from the receptor and is released into the circulation [124].

The homeostasis of iron in the body is regulated to ensure a sufficient portion of iron for the many essential functions in which it is involved and to avoid excess or a lack of iron [126]. When more iron is needed, the body increases intestinal absorption, resulting in the release of this metal by macrophages and other cells that store it. On the other hand, absorption is inhibited when there is too much iron, and storage is increased to prevent all possible toxic effects. All the steps necessary to maintain homeostasis are regulated at the systemic and cellular levels [122].

The regulation of plasma iron concentrations is made by hepcidin, a peptide hormone with 25 amino acids, which also plays a crucial role in inflammation and immune responses to infection, preventing microorganisms from using iron sources for their growth and proliferation [127,128]. Hepcidin is produced primarily in hepatocytes, although it is also synthesized in smaller amounts by other cell types, such as neutrophils, monocytes, lymphocytes, adipocytes, and pancreatic and renal β cells, and is excreted by urine [123]. It is a regulatory hormone that acts as an inhibitor of iron flux from tissues to plasma by binding to FPN1. This binding leads to the internalization and degradation of FPN1, reducing its levels and consequently reducing iron absorption from the diet and its release by macrophages and hepatocytes [129].

Different signals regulate hepcidin expression via the response to circulating iron levels, erythropoietic activity, and the inflammatory response [129]. The first mechanism involves several molecules that function as “sensors” of iron, such as the homeostatic iron regulator (HFE), transferrin receptor 1 (TfR1), and transferrin receptor 2 (TfR2) [122].

HFE is a protein-like major histocompatibility complex (MHC) class I-type protein interacting with the transferrin receptors TfR1 and TfR2. When iron homeostasis is present, HFE is located on the cell surface of the hepatocyte and is bound to TfR1, which delivers Tf-(Fe^3+^)_2_ to most cells. However, when circulating iron levels increase, Tf-(Fe^3+^)_2_ gains high affinity for TfR1. Since the binding sites of TfR1 for HFE and Tf-(Fe^3+^)_2_ are overlapping and the affinity of TfR1 is higher for Tf-(Fe^3+^)_2_ than for HFE, the latter dissociates from TfR1 and becomes available to associate with TfR2, increasing the expression of hepcidin [123,129,130].

The liver is the main iron storage organ. One-third of the body’s total iron is in hepatocytes, portal vessels, sinusoidal mesenchymal cells, and reticuloendothelial cells. The liver also plays an essential role in iron metabolism, as it is here that Tf (its main carrier protein) and Ft (the main storage protein) are synthesized [56].

CHC is frequently associated with iron overload, with about 10–42% of individuals showing hepatic iron accumulation. In addition, 20–35% of patients with CHC have increased levels of transferrin saturation (TS), serum iron (Fe), and serum Ft [55], and these parameters correlate significantly and directly with liver fibrosis [118].

Indirect evidence based on serum measurements of iron stores supports that excess iron may aggravate hepatic necroinflammatory activity in chronic viral hepatitis. Iron overload may increase fibrosis because of oxidative stress upon lipid peroxidation with subsequent production of free oxygen radicals. Consequently, it has been hypothesized that hepatic iron overload may influence the progression of CHC and the severity of disease manifestations [131].

The mechanism by which iron accumulates in the liver of those patients has not yet been established, and it is unclear if this accumulation is the primary cause or a secondary result of liver injury [132].

During hepatic inflammation, damaged hepatocytes can release iron into the surrounding environment [56]. Evidence indicates that elevated hepatic iron concentrations may exacerbate necroinflammatory activity in CHC and promote the progression of hepatic fibrosis [131,133]. Excessive iron deposition in CHC contributes to oxidative stress and subsequent liver injury, leading to enhanced hepatocyte necrosis and apoptosis, activation of hepatic stellate cells, and stimulation of fibrogenic pathways characterized by increased actin and collagen synthesis [134].

Iron accumulation in CHC is thought to result primarily from hepatocellular injury, during which infected hepatocytes release hemosiderin that is subsequently phagocytosed by KCs. Additional mechanisms have been proposed, including hepatocyte regeneration, cytokine-mediated modulation of iron metabolism, alterations in iron uptake related to chronic necroinflammation, and intrahepatic vascular shunting [135].

Ft levels serve as a key biomarker for assessing the severity of hepatic injury and the extent of fibrosis-associated necroinflammatory activity. Like iron, ferritin is liberated from injured hepatocytes during inflammatory states, with its serum concentration correlating positively with the intensity of inflammation [56]. The same study also identified decreased total iron-binding capacity (TIBC) in patients with advanced fibrosis. Inflammatory processes are frequently accompanied by systemic disturbances in iron homeostasis, primarily driven by elevated hepcidin expression, which regulates both intestinal iron absorption and macrophage iron release [136,137].

**Figure 5:**
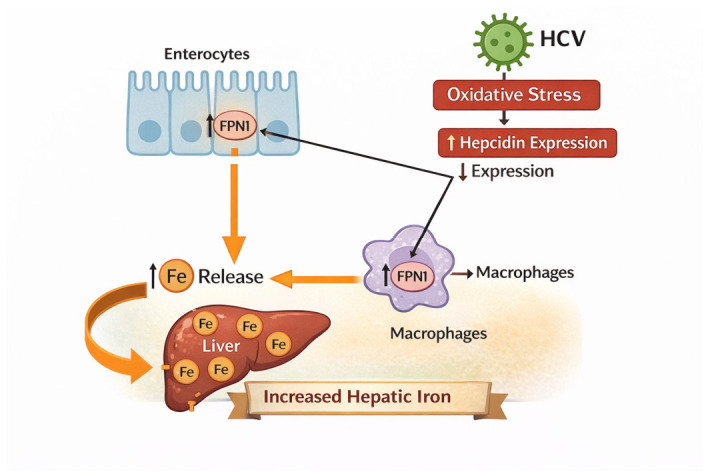
Dysregulation of iron homeostasis in the presence of HCV. HCV: hepatitis C virus; FPN1: ferroportin; Fe: iron.

In the presence of HCV infection, hepcidin transcription is suppressed through oxidative stress-mediated mechanisms, resulting in decreased hormone expression and, consequently, increased levels of FPN1 in enterocytes and reticuloendothelial macrophages. This dysregulation enhances intestinal iron absorption and promotes iron release from macrophages, leading to systemic iron overload [117,133] (Figure 5). Moreover, in viral hepatitis, the rise in hepatic iron may also represent a defensive host response, whereby liver cells accumulate iron to restrict pathogen access and inhibit their proliferation [138]. Notably, the hepcidin–ferritin ratio has been shown to be significantly lower in individuals with HCV compared to uninfected individuals or those with hepatitis B virus [117].

HCV clearance with DAAs has been shown to normalize serum iron parameters, and this occurs soon after antiviral treatment, suggesting a direct effect of the virus on iron metabolism [139,140]. On the other hand, this normalization has a durable impact, meaning that abnormalities of iron metabolism in CHC may also be a consequence of viral infection, such as inflammation, that is resolved with HCV elimination [141].

### 4.5. Oxidative Stress

Chronic HCV infection is closely linked to oxidative stress—defined as an imbalance between reactive oxygen/nitrogen species (ROS/RNS) and the body’s antioxidant defenses. Multiple viral proteins—including core proteins, NS3, NS4B, and NS5A—interfere with mitochondrial function, induce nicotinamide adenine dinucleotide phosphate (NADPH) oxidases, and deplete cellular antioxidants such as GSH, resulting in increased ROS production [41]. The core protein, for instance, goes to and accumulates in the mitochondria, impairing electron transport at complexes I and III, altering calcium uptake, and promoting ROS accumulation. In transgenic mice expressing core proteins, hepatic levels of 8-oxo-deoxyguanosine (8-oxodG), a marker of oxidative DNA damage, are significantly elevated compared to controls [141]. NS5A further exacerbates oxidative stress via calcium release from the endoplasmic reticulum, activating ROS-dependent signaling cascades and contributing to the pro-inflammatory milieu. NS3 activates NADPH oxidase 2 (Nox2) in immune cells, increasing reactive oxygen species (ROS) production and promoting hepatocyte apoptosis [142].

These mechanisms and the pro-inflammatory milieu, in which the liver is in a chronic state of immune activation and inflammation due to viral proteins, oxidative stress, and ongoing immune responses, cause sustained oxidative injury that promotes viral persistence, immune evasion, inflammation, liver cell damage, and fibrosis and increases the risk of progression to cirrhosis or liver cancer.

ROS play a central role in activating HSCs and fibrogenesis. They stimulate TGF-β, the primary profibrotic cytokine, leading to excess extracellular matrix deposition and fibrosis. ROS-generated lipid peroxidation products, such as malondialdehyde (MDA) and 4-hydroxynonenal (HNE), induce hepatocyte apoptosis and inflammation, further activating HSCs in a self-perpetuating cycle [143]. A study from 2003 showed higher values of MDA in patients with CHC and a decrease after HCV clearance [144].

Oxidative stress disrupts insulin signaling (via altered Akt/PP2A), inducing insulin resistance, upregulating lipogenic transcription factor SREBP 1, and promoting hepatic fat accumulation (steatosis). Steatosis exacerbates oxidative damage and fibrogenesis, shaping a metabolic–inflammatory phenotype often seen in HCV patients—sometimes labeled “HCV-associated dysmetabolic syndrome” [145].

Recent studies show that successful DAA therapy leads to significant improvements in redox balance beyond viral clearance. In one cohort (n = 196), serum 8 oxodG levels declined significantly four weeks into therapy; MDA and HNE levels dropped by the end of treatment and stayed low at 12 weeks post-treatment [146]. Similar findings include normalization of oxidized LDL, with levels correlating to improvements in liver stiffness, especially two years post-SVR [147].

DAA therapy enhances the activity of antioxidant enzymes—superoxide dismutase (SOD), catalase (CAT), and glutathione peroxidase (GSH Px)—in circulating immune cells by treatment completion and persists through SVR12 [146]. Additionally, the dynamic thiol–disulfide system lowers disulfide levels and increases native thiols, indicating restored redox capacity [143].

In summary, viral proteins disrupt mitochondrial function and activate ROS-generating enzymes, establishing oxidative stress as a core pathogenic mechanism in HCV. ROS cause fibrosis, inflammation, and steatosis and indirectly promote carcinogenesis through DNA damage and mutagenesis.

SVR leads to decreased oxidative damage, enhanced antioxidant defenses, and downregulation of profibrotic signaling. Antioxidant recovery may improve tissue regeneration, delay fibrosis progression, and reduce risks of cirrhosis, hepatocellular carcinoma, and cardiovascular diseases. Longitudinal studies are needed to assess long-term outcomes.

In conclusion, oxidative stress is integral to HCV pathogenesis and mediates key progression pathways in liver injury. DAAs not only eliminate the virus but also initiate a cascade of biochemical reversals that restore redox balance, suppress fibrogenic signaling, and foster long-term hepatic repair mechanisms that pave the way for improved clinical outcomes.

### 4.6. Oxidative Stress, Iron, and Aconitase: A Pathogenic Triangle

CHC virus infection is associated with sustained hepatic inflammation and altered iron metabolism, both of which contribute to oxidative stress and mitochondrial dysfunction. A key player in this cascade is aconitase, an iron–sulfur enzyme with both metabolic and regulatory roles, and it is particularly susceptible to oxidative inactivation.

In CHC, hepatic iron overload is a common feature due to increased intestinal absorption, impaired hepcidin regulation, and hepatocellular retention. Iron acts as a pro-oxidant by participating in the Fenton reaction, where Fe^2+^ reacts with hydrogen peroxide (H_2_O_2_) to produce highly reactive hydroxyl radicals (•OH), amplifying ROS levels in hepatocytes [148]. Aconitase exists in two main isoforms: mitochondrial aconitase (m-aconitase), which is involved in the tricarboxylic acid (TCA) cycle, catalyzing citrate to isocitrate and cytosolic aconitase (c-aconitase) which functions as a bifunctional protein.Under oxidative stress or iron deficiency, it loses its Fe–S cluster (CIS) and acts as an iron regulatory protein (IRP1), binding to iron-responsive elements (IREs) on mRNAs involved in iron metabolism. In HCV-infected hepatocytes, oxidative stress leads to the inactivation of both aconitase isoforms. When m-aconitase is active, citrate is isomerized to isocitrate by aconitase; then, isocitrate is oxidatively decarboxylated to α-ketoglutarate with concomitant generation of NADH. However, when it is inactivated by ROS, citrate accumulates, and it is exported to the cytosol and cleaved into acetyl-CoA and oxaloacetate, with the first one serving as the substrate for fatty acid synthesis. In parallel, cytosolic reactions (e.g., via a malic enzyme or the pentose phosphate pathway) produce reduced nicotinamide adenine dinucleotide phosphate (NADPH), which is essential as a reducing agent in lipogenic/anabolic processes [149]. Regarding c-aconitase, in the presence of ROS, it shifts to its IRP1 form, leading to increased expression of TfR1 and suppressed Ft translation, promoting intracellular iron uptake, and reducing iron storage—thereby exacerbating iron-mediated ROS production in a vicious cycle [150,151]. This iron/ROS/aconitase loop contributes to mitochondrial dysfunction; ATP depletion; activation of HSCs via ROS and TGF-β pathways; DNA damage; oncogenic transformation through lipid peroxidation products, such as 4-HNE and MDA; and progression to fibrosis, cirrhosis, and HCC, even after viral clearance with DAAs [152,153,154].

**Figure 6:**
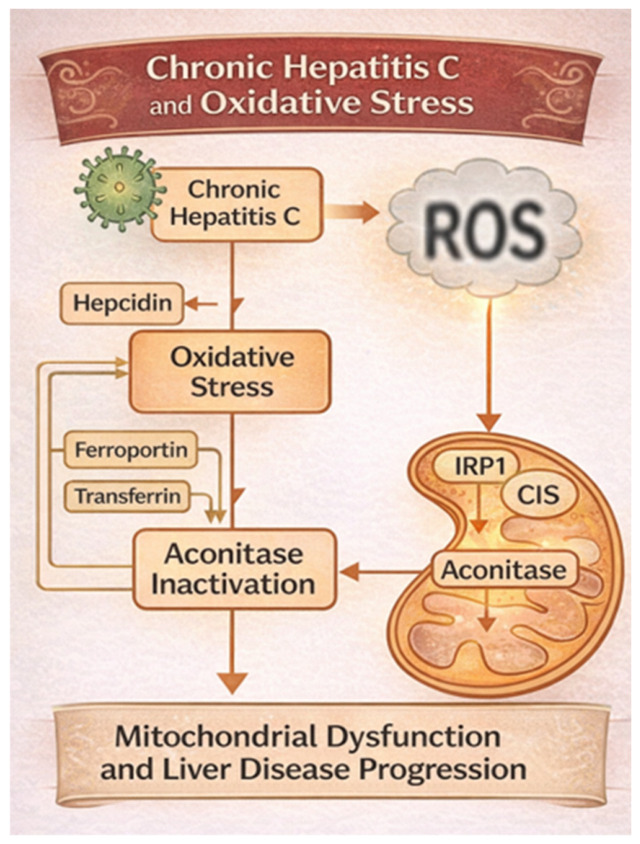
Oxidative stress, iron and aconitase in chronic hepatitis C. ROS: reactive oxygen species; IRP1: iron regulatory protein; CIS: cluster iron–sulfur.

In CHC, iron overload synergizes with HCV-induced oxidative stress to inactivate aconitase enzymes, disturbing both mitochondrial energy production and iron regulation. This pathogenic interaction sustains oxidative injury, promotes fibrogenesis, and facilitates hepatocarcinogenesis. Understanding this interplay may provide a mechanistic basis for targeting iron metabolism and redox pathways in adjunctive therapies for HCV-related liver disease (Figure 6).

### 4.7. Glucose Metabolism

The liver is also responsible for maintaining glucose metabolism, storing glucose, and producing endogenous glucose from glycogen stores in the liver or via gluconeogenesis. These activities contribute to preserving normal blood glucose levels [155].

The insulin receptor is a tyrosine kinase receptor consisting of two extracellular α subunits carrying insulin binding sites and two transmembrane β subunits with tyrosine kinase activity, and it is involved in intracellular signaling and linked by disulfide bonds. The binding of insulin to its receptor activates the PI3K/AKT signaling pathway, stimulating glucose transport across the cell membrane through glucose transporter 4 (GLUT4) and increasing the rate of glucose influx.

HCV alters glucose metabolism by inducing inflammatory cascades and promoting insulin resistance.

Glucose transport to hepatocytes is done by glucose transporter 2 (GLUT2), which is downregulated by the HCV core protein. On the other hand, HCV infection promotes the overproduction of TNF-α and other cytokines, such as IL-6, that inhibit the insulin receptor substrate (IRS) and phosphatidylinositol 3 kinase (PI3K). This alteration in intracellular insulin signaling could block the activation of GLUT4 and reduce cells’ glucose uptake [156]. Patients with chronic hepatitis and cirrhosis showed decreased hepatic insulin extraction due to liver damage. The progressive loss of functional hepatocytes associated with fibrosis reduces hepatic insulin uptake and glucose metabolism, ultimately leading to insulin resistance (IR) [157]. GAGs, as key components of the ECM, which undergo major alterations during the fibrotic process, actively contribute to the formation and maintenance of the fibrotic ECM by modulating the activity of profibrotic cytokines and growth factors, such as TGF-β and fibroblast growth factor (FGF). Their accumulation increases tissue stiffness and perpetuates a profibrotic microenvironment. These structural and functional changes in the extracellular matrix can impair insulin signaling by disrupting insulin receptor accessibility, altering mechano-transduction, and sustaining chronic inflammation. Consequently, GAGs not only serve as key structural components of fibrosis but also contribute to the development of insulin resistance in fibrotic tissues, particularly in the liver [158,159].

Higher HCV RNA levels were associated with higher homeostatic model assessment for insulin resistance (HOMA-IR), and its improvement was correlated with a decrease in viral load [160,161,162]. Moreover, regarding the changes in glucose metabolism after DAA therapy, a study showed that clearance of HCV with antiviral treatment resulted in the restoration of insulin sensitivity [163].

In the presence of liver damage, less insulin is absorbed by the hepatocyte and degraded, causing a situation of chronic hyperinsulinemia. Advanced fibrosis and liver cirrhosis may be accompanied by IR, causing inefficiency of the liver in metabolizing excess glucose. As a result, blood glucose levels increase.

**Figure 7:**
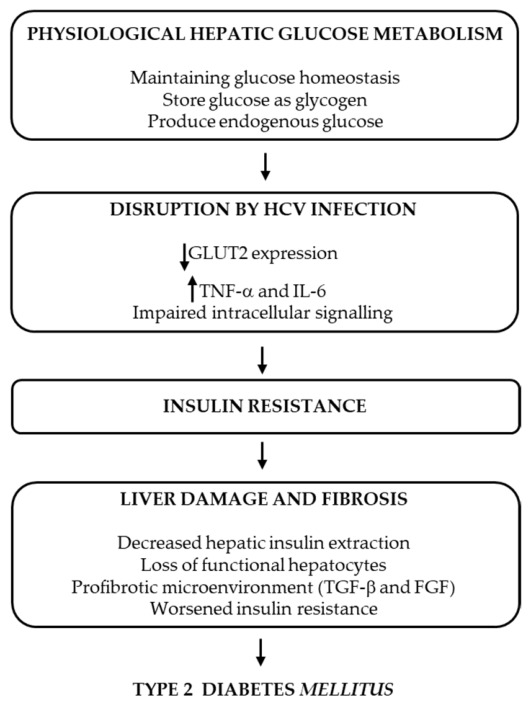
HCV-induced alterations in glucose metabolism and insulin resistance. GLUT2: glucose transporter 2; TNF-α: tumor necrosis factor-alpha; IL-6: interleukin-6; TGF-β: transforming growth factor; FGF: fibroblast growth factor.

Insulin resistance, impaired glucose tolerance, and T2DM are frequent extrahepatic manifestations of HCV [44,121,164], and some studies have shown a positive correlation between T2DM, IR, and liver fibrosis progression in patients with chronic HCV infection [44,134,165]. Other studies found that the development of IR in patients with chronic HCV can occur early in the course of the disease, and this effect appears to be independent of body weight, the stage of liver disease, and the presence or absence of overt diabetes [134,166] (Figure 7).

## 5. Host Genetic Background in Liver Disease Progression and Hepatocellular Carcinoma Risk After DAA Therapy

Chronic infection with hepatitis C virus promotes progressive fibrosis and cirrhosis, laying the groundwork for HCC. A study published in 2011 demonstrated a reduced risk of HCC in patients with cirrhosis who achieved SVR following interferon-based therapy [167]. In the era of DAAs, although SVR is typically achieved and hepatic inflammation is substantially reduced, a residual risk of HCC persists, particularly among individuals who have already developed advanced fibrosis or cirrhosis prior to viral eradication [168].

Recent genetic investigations focus on host polymorphisms that predispose patients to fibrosis progression and HCC even after a sustained virologic response. Early Genome-Wide Association Studies (GWAS) and candidate gene studies have highlighted variants in *PNPLA3* (rs738409), *MBOAT7* (rs641738), *TM6SF2* (rs58542926) and *GCKR*, which are aggregated into a lipotoxicity-based polygenic risk profile that predicts the genetic risk score (GRS) for hepatic fat accumulation, steatosis, fibrosis progression, and HCC risk, even after HCV elimination with DAAs [163]. In a cohort of DAA-treated cirrhotic patients, a GRS above the threshold (>0.597) was independently associated with a ~2.3 fold increased hazard ratio (HR) of de novo HCC during a median follow-up of 43 months [169,170]. *PNPLA3* (rs738409) resides at chromosome 22q13.31 and encodes patatin-like phospholipase domain-containing protein 3, which regulates triglyceride breakdown in hepatocytes. The I148M variant (G allele) impairs lipolysis, promoting fat accumulation, accelerating fibrosis, and increasing HCC risk in cirrhotic cohorts [171]. *MBOAT7* (rs641738) is located on chromosome 19q13.42 and encodes a membrane O-acyltransferase involved in phospholipid remodeling and inflammatory lipid mediator production. The T allele is associated with reduced gene expression, increased fibrosis, steatosis, and HCC risk (OR: 1.65–2.1 in NAFLD and HCV contexts). *TM6SF2* (rs58542926) resides at chromosome 19p13.11 and encodes a protein essential for VLDL secretion. The E167K variant limits lipid export from hepatocytes, promoting intracellular triglyceride retention. It is associated with steatosis and contributes to fibrosis and elevated HCC risk, particularly when combined with *PNPLA3* and *MBOAT7* risk alleles. *GCKR* (rs1260326) sits on chromosome 2p23.3 and encodes the glucokinase regulatory protein, which modulates hepatic glucose uptake. Its variant alters glucokinase inhibition, boosting glycolytic flux and de novo lipogenesis, thus promoting hepatic fat deposition and increasing HCC susceptibility when included in polygenic models. This body of evidence indicates that risk variants in *PNPLA3*, *MBOAT7*, *TM6SF2*, and *GCKR*—genes encoding proteins involved in lipid metabolism, phospholipid remodeling, VLDL secretion, and glucose regulation—collectively constitute a hepatic fat-related genetic signature that improves the prediction of fibrosis and HCC in both the NAFLD and post-HCV cure contexts. Recent studies highlight the value of such polygenic risk scores for personalized risk assessment and integrated prognostication in liver disease management.

Other candidate loci linked to post-SVR HCC include *MICA* (rs2596542), *DEPDC5*, *TLL1* (rs17047200), and *HLA DQB1* variants, which are most notably identified in Japanese cohorts [172]. The *MICA* gene, located at 6p21.33, encodes a stress-induced ligand for the NK-cell receptor NKG2D; the promoter SNP rs2596542 reduces *MICA* expression and is linked to accelerated fibrosis progression, indirectly raising the risk of HCC [173]. The gene *DEPDC5*, located at 22q12.2–q12.3, encodes a regulator of mTORC1 signaling; an intronic variant (rs1012068) has been tied to increased HCC susceptibility in GWAS data; however, this result is not consistently replicated [174]. *TLL1*, located at 4q32.3, encodes a metalloprotease involved in extracellular matrix remodeling; its intronic SNP, rs17047200, showed a strong association with HCC risk post-HCV clearance, likely by influencing fibrogenesis [175]. Finally, *HLA-DQB1*, located at 6p21.32, encodes the β-chain of class II HLA-DQ molecules; certain alleles confer elevated HCC risk, possibly via altered capacity for antigen presentation and immune surveillance [176]. These observations underscore the importance of germline genetic loci in modulating fibrosis progression and residual HCC risk, even after viral eradication, by affecting immune surveillance, fibrotic remodeling, and mTOR signaling.

Studies of *IFNL3/IFNL4* (IL28B) locus variants, which are originally associated with HCV clearance, have extended to fibrosis and HCC risk. Specific nonresponder genotypes of rs12979860 have been linked to greater hepatic inflammation, accelerated fibrosis, and in some cohorts, an earlier or higher incidence of HCC after SVR [177]. Furthermore, emerging multi-omics studies, including those involving genotyping, transcriptomics, and epigenetic profiling, demonstrate that mutations in the telomerase reverse transcriptase (*TERT)* promoter and oncogenic pathways (Wnt/β catenin, p53, and PI3K/Akt/mTOR), as well as persistent histone modifications, such as upregulation of sphingosine Kinase 1 (SPHK1), remain detectable in post-SVR liver tissue and contribute to residual oncogenic drive [178]. Genetic variants and their functional impact on HCC development in CHC and post-SVR settings are described in Table 1.

By integrating host genetic data with clinical markers (e.g., liver stiffness), personalized risk stratification models can better identify patients at high residual HCC risk after SVR [171]. In the era of precision medicine, such host genetic profiling may help tailor surveillance intensity by identifying high-risk individuals who would benefit from more frequent ultrasound or Magnetic Resonance Imaging monitoring, even after HCV cure. It may also support prioritization of targeted preventive strategies—such as lifestyle modifications or emerging chemopreventive agents—for individuals with a lipotoxicity-related genetic predisposition. Finally, incorporating genetic profiling into clinical practice may enhance prognostic counseling, refine long-term outcome prediction, and guide personalized follow-up planning—ultimately empowering both patients and clinicians to make informed decisions.

## 6. Persistent Fibrosis and Carcinogenesis After HCV Eradication: The Role of Epigenetic Memory in Sustained Hepatocellular Carcinoma Risk

Although eradication of HCV infection with direct acting antivirals (DAAs) markedly reduces inflammation, halts viral replication, and significantly decreases the incidence of HCC, it does not fully eliminate the risk of HCC in all patients, especially those with advanced fibrosis or cirrhosis prior to treatment [179]. This residual risk reflects complex and incompletely reversible alterations in liver biology that extend beyond viral clearance.

One of the major mechanisms underlying persistent disease progression and carcinogenesis after a sustained virologic response is the long-lasting reprogramming of the epigenome induced by chronic HCV infection. Epigenetic modifications—including DNA methylation, post-translational histone modifications, and non-coding RNA regulation—orchestrate gene expression programs that are essential for normal hepatic homeostasis. Chronic HCV infection disrupts these mechanisms, leading to genome-wide changes that alter chromatin accessibility and the transcriptional output of cancer-related pathways [180,181]. These changes are not solely dependent on the continued presence of viral RNA or proteins but rather represent stable alterations that persist after viral eradication.

Genome-wide analyses have demonstrated that specific histone marks, such as H3K27 acetylation, remain altered in liver tissue even after SVR and are associated with dysregulated transcriptional programs linked to cancer risk [180]. Correspondingly, DNA methylation profiling in post-SVR livers shows that many differentially methylated regions induced by chronic HCV remain after therapy, affecting the expression of transcription factors and their downstream targets implicated in apoptosis and inflammatory signaling—processes closely tied to carcinogenesis [181].

Persistent epigenetic “scars” have also been observed in immune cells. For example, regulatory T cells from patients successfully treated for chronic HCV retain epigenetic and transcriptomic features consistent with an activated and inflammatory phenotype, even after DAA-induced viral clearance [180]. Such immune cell reprogramming may contribute to a pro-tumorigenic microenvironment that facilitates tumor initiation or progression despite the absence of active infection.

These observations support the concept of an HCV-induced epigenetic memory: a stable, virus driven imprint on the host epigenome that continues to influence gene expression and cellular behavior after the virus itself has been eliminated. Epigenetic memory is widely recognized in other contexts (e.g., cancer and chronic inflammation) and can perpetuate aberrant regulatory states long after the inciting event has been resolved. In the setting of HCV, this may contribute to the incomplete reversibility of disease processes such as fibrogenesis, as well as the ongoing risk of HCC despite an SVR.

Fibrosis and cirrhosis can partially regress following successful viral eradication, and improvements in clinical outcomes, including reduced portal hypertension and decreased liver related mortality, have been reported [179]. However, the degree of fibrosis regression varies between patients and is influenced by the baseline severity of liver disease, host factors, and possibly the persistence of epigenetic alterations that sustain fibrogenic signaling pathways. These pathways involve activated hepatic stellate cells and profibrotic cytokines, which may remain active due to epigenetically maintained gene expression patterns even after the SVR.

**Figure 8:**
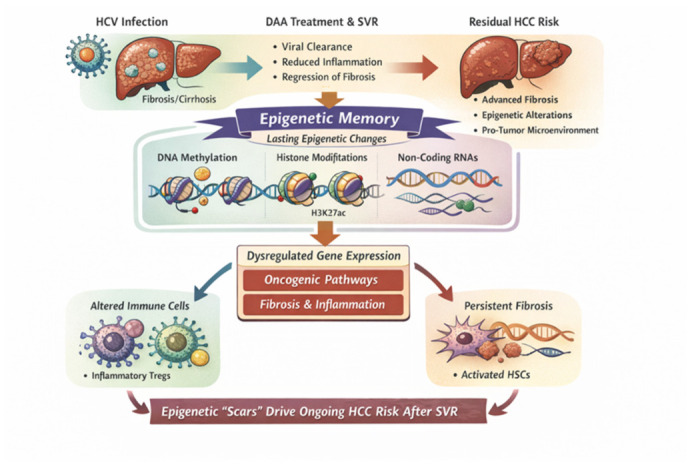
Epigenetic memory and persistent risk of hepatocellular carcinoma after a sustained virologic response in HCV infection. HCC: hepatocellular carcinoma; SVR: sustained virologic response; Tregs: regulatory T cells; H3K27 ac: H3K27 acetylation.

Collectively, these data emphasize that epigenetic reprogramming represents a mechanistic link between chronic HCV infection and long-term liver disease outcomes, including HCC risk. Persistent epigenetic signatures, detectable years after viral cure, reveal that the host epigenome retains a molecular memory of the disease. This memory may underline the continued activation of oncogenic pathways, suboptimal reversal of fibrosis, and ongoing carcinogenic risk in certain populations of SVR patients (Figure 8). Elucidating these mechanisms further could inform the development of biomarkers to stratify post-SVR risk and epigenetic therapies aimed at reversing these enduring changes.

## 7. Conclusions

CHC virus infection orchestrates a complex interplay between disturbances in iron, lipid, and glucose metabolism; oxidative stress; and immune-mediated acute-phase responses, collectively driving progressive liver injury.

HCV infection leads to suppression of hepatic hepcidin synthesis, which is mediated by oxidative stress and inhibition of transcriptional regulators, resulting in increased intestinal iron absorption, macrophage iron retention, and hepatic iron overload. Elevated intracellular Fe^2+^ expression promotes the Fenton reaction, generating hydroxyl radicals that augment ROS and leading to lipid peroxidation, mitochondrial dysfunction, DNA damage, and, ultimately, ferroptosis, a type of iron-dependent regulated cell death characterized by the accumulation of peroxidizable lipids and the failure of cellular antioxidant defense mechanisms [148,178,182].

Concurrently, HCV disrupts lipid metabolism by enhancing de novo lipogenesis and impairing β-oxidation. The resulting hepatic steatosis and accumulation of lipid peroxidation products exacerbate oxidative injury and stimulate profibrogenic pathways via TGF-β, leading to the activation of hepatic stellate cells and collagen deposition [183,184].

At the same time, HCV impairs glucose metabolism by promoting IRS-1 degradation and elevating pro-inflammatory cytokines, fostering IR. IR further drives lipogenesis and steatosis, intensifying inflammation, oxidative damage, and the risk of T2DM [185].

Chronic inflammation also affects acute-phase proteins, particularly hp, which binds free hemoglobin to prevent heme-mediated oxidative damage. Liver damage deregulates hp isoforms and reduces total serum hp levels, allowing free hemoglobin and heme to accumulate and promote additional ROS formation [186,187].

A central mechanism in this pathogenic network involves aconitase inactivation. Under oxidative stress, m-aconitase loses its [4Fe–4S] cluster, impairing ATP synthesis and leading to the accumulation of citrate which culminates in fatty acid synthesis. Meanwhile, cytosolic aconitase transitions to IRP1, binding to IREs on mRNAs to stabilize TfR1 transcripts and repress Ft translation. This shift increases iron uptake and decreases storage, reinforcing Fe-mediated ROS generation. Ongoing ROS accumulation fosters mitochondrial dysfunction, DNA injury, HSC activation, and oncogenic transformation, even following HCV eradication with DAAs [22,183].

**Figure 9:**
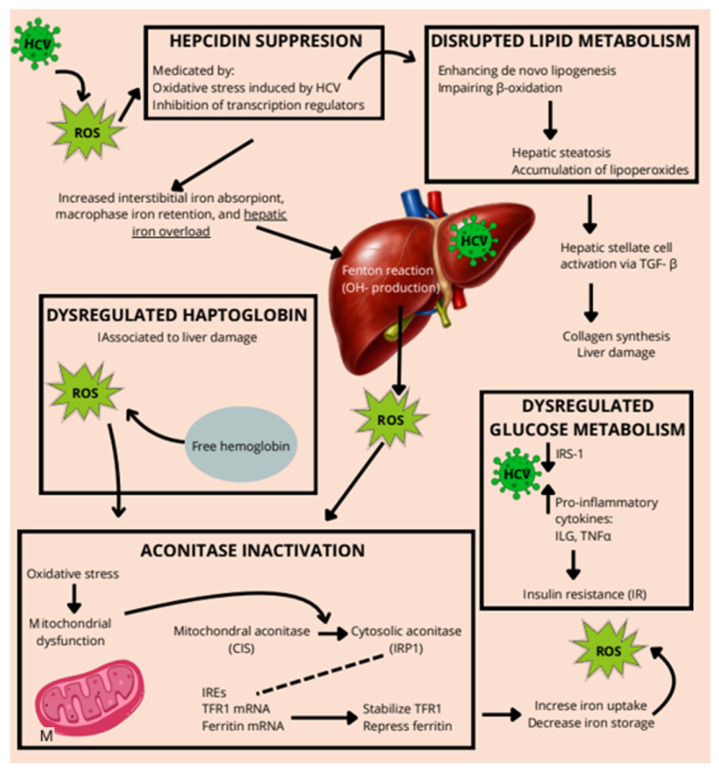
Chronic hepatitis C infection and associated pathogenesis. HCV: hepatitis C virus; ROS: reactive oxygen species; TGF-β: tumor growth Factor-β; TNF-α: tumor necrosis factor-α; IRP1: iron regulatory protein; IREs: iron-responsive elements; IRS-1: insulin receptor substrate; CIS: cluster iron–sulfur; TFR1: transferrin receptor 1.

Thus, CHC induces a pathogenic cycle whereby iron overload, disrupted glucose and lipid metabolism, acute-phase protein dysregulation, and immunologic activation interlink to promote mitochondrial injury, genomic instability, and fibrogenesis. Targeting this nexus, through modulation of hepcidin, iron chelation, antioxidative therapies, metabolic interventions, and acute-phase protein support, may offer promising adjunctive strategies to improve long-term outcomes in patients cured of HCV (Figure 9).

Moreover, recent host genetic studies have begun to reveal the critical role of genetic variants in predicting fibrosis progression and persistent HCC risk even after DAA-mediated HCV clearance. Together with insights into somatic mutations and epigenetic persistence, these discoveries pave the way for individualized surveillance, risk-adapted therapy, and better prognostic stratification. Ultimately, integrating genetic, epigenetic, and clinical data could improve outcomes for patients cured of hepatitis C by guiding precision medicine approaches, reducing HCC incidence, and optimizing long-term survival.

## Figures and Tables

**Table 1:** Genetic variants and their functional impact on hepatocellular carcinoma (HCC) development in chronic hepatitis C and post-SVR settings.

Gene	Variant (SNP/Locus)	Encoded Protein Function	Polymorphism Mechanistic Effect of Mutation	Impact on Liver Pathology and HCC Risk	**References**
*PNPLA3*	rs738409 (I148M, Chr 22q13.31)	Patatin-like phospholipase domain-containing protein 3 Regulates triglyceride hydrolysis in hepatocytes	G (I148M) allele Impairs triglyceride breakdown, causing lipid accumulation	Promotes steatosis, accelerates fibrosis, and increases HCC risk, especially in cirrhotic patients	[169,170,171]
*MBOAT7*	rs641738 (Chr 19q13.42)	Membrane O-acyltransferase Involved in phospholipid remodeling and inflammatory lipid mediator synthesis	T allele Reduces gene expression and alters phospholipid metabolism	Associated with higher fibrosis, steatosis, and HCC risk (OR: 1.65–2.1 in NAFLD and HCV)	[169,171]
*TM6SF2*	rs58542926 (E167K, Chr 19p13.11)	Transmembrane 6 superfamily member 2 Regulates VLDL secretion	E167K variant Impairs lipid export from hepatocytes, leading to triglyceride retention	Enhances steatosis and fibrosis; augments HCC risk, particularly in presence of PNPLA3 and MBOAT7 variants	[169,171]
*GCKR*	rs1260326 (Chr 2p23.3)	Glucokinase regulatory protein Modulates hepatic glucose uptake	Variant Decreases glucokinase inhibition, increases glycolysis and de novo lipogenesis	Promotes hepatic fat accumulation and contributes to HCC susceptibility in polygenic models	[169,171]
*MICA*	rs2596542 (Chr 6p21.33)	MHC class I polypeptide-related sequence A Ligand for NK-cell receptor NKG2D	Promoter SNP Decreases MICA expression and weakens NK-cell activation	Enhances fibrosis progression and indirectly raises HCC risk post-SVR	[172,173]
*DEPDC5*	rs1012068 (Chr 22q12.2–q12.3)	DEP domain–containing protein 5 Regulator of mTORC1 signaling	Intronic variant May alter mTOR pathway regulation	Associated with higher HCC susceptibility, though replication inconsistent	[172,174]
*TLL1*	rs17047200 (Chr 4q32.3)	Tolloid-like 1 metalloprotease Involved in ECM remodeling	Intronic variant Influences fibrogenic activity and ECM deposition	Strongly linked with HCC development after HCV clearance	[172,175]
*HLA-DQB1*	Various alleles (Chr 6p21.32)	HLA class II β-chain Antigen presentation	Variant alleles Alter antigen presentation and immune surveillance	Associated with increased HCC risk due to impaired immune monitoring	[172,176]
*IFNL3/IFNL4 (IL28B)*	rs12979860	Interferon lambda ¾ Mediates antiviral immune response	Non-responder genotype Linked to persistent inflammation and fibrosis	Associated with greater hepatic inflammation, accelerated fibrosis, and increased HCC incidence post-SVR	[177]
*TERT* promoter	—	Telomerase reverse transcriptase Maintains telomere integrity	Promoter mutations Enhance TERT expression	Promote oncogenic transformation and sustained hepatocyte proliferation	[178]
*SPHK1*	—	Sphingosine kinase 1 Modulates sphingolipid signaling	Epigenetic upregulation (histone modification) Increases oncogenic signaling	Maintains pro-tumorigenic environment in post-SVR liver tissue	[178]
Wnt/β-catenin, p53, PI3K/Akt/mTOR pathways	—	Oncogenic and tumor-suppressor signaling pathways	Somatic or epigenetic alterations Activate oncogenic signaling	Drive hepatocarcinogenesis and residual HCC risk post-viral eradication	[178]

## Data Availability

No new data were created or analyzed in this study. Data sharing is not applicable to this article.

## Abbreviations

ALT	alanine aminotransferase
Apo B	apolipoprotein B
Apo C1	apolipoprotein C1
Apo E	apolipoprotein E
apo-Tf	apo-transferrin
AST	aspartate aminotransferase
BMI	body mass index
c-aconitase	cytosolic aconitase
CAT	catalase
CE	cholesterol esters
CHC	chronic hepatitis C
CKs	Kupffer cells
cLDs	cytosolic lipid droplets
CLDN1	claudin-1
CTL	cytotoxic T lymphocyte
DAAs	direct acting antivirals
DAG	diacylglycerol
DCYTB	duodenal cytochrome b
DGAT1	diacylglycerol O-acetyltransferase
DMT1	divalent metal transporter 1
DNA	deoxyribonucleic acid
ECM	extra cellular matrix
ER	endoplasmic reticulum
Fe	free ion
Fe^2+^	iron ferrous form
Fe^3+^	iron ferric form
FGF	fibroblast growth factor
FPN1	ferroportin
Ft	ferritin
GAGs	glycosaminoglycans
GB-A	hepatitis G virus A
GB-B	hepatitis G virus B
GB-C	hepatitis G virus C
GLUT2	glucose transporter 2
GLUT4	glucose transporter 4
GRS	genetic risk score
GSH	glutathione
GSH Px	glutathione peroxidase
GWAS	genome wide association study
HCC	hepatocellular carcinoma
HCP1	haem carrier protein 1
HCV	hepatitis C virus
HDL	high density lipoprotein
HFE	human homeostatic iron regulator
HNE	4-hydroxynonenol
HO-1	hemeoxygenase-1
HOMA-IR	homeostatic model assessment for insulin resistance
Hp	haptoglobin
Hp gene	haptoglobin gene
HR	hazard ratio
HSCs	hepatic stellate cells
H_2_O_2_	hydrogen peroxide
IFNγ	interferon gamma
IL-10	interleukin 10
IL-2	interleukin 2
IL-6	interleukin 6
IR	insulin resistance
IREs	iron-responsive elements
IRP1	iron regulatory protein
IRS	insulin receptor substrate
KCs	Kupffer cells
LDL	low-density lipoprotein
LDLox	oxidized low-density lipoprotein
LDLR	low-density lipoprotein receptor
LPL	lipoprotein lipase
LVPs	lipoviral particles
Mac-1	macrophage-1 antigen
m-aconitase	mitochondrial aconitase
MDA	malondialdehyde
MHC	major histocompatibility complex
MMPs	matrix metalloproteinases
NOX2	NADPH oxidase 2
NPC1L1	Niemann-Pick C1-Like 1
NS2	non-structural protein 2
NS4A	non-structural protein 4A
NS4B	non-structural protein 4B
NS5A	non-structural protein 5A
NS5B	non-structural protein 5B
ORF	open reading frame
•OH	hydroxyl radicals
PC	phosphatidylcholines
p7	non-structural protein p7
PI3K	phosphatidylinositol 3 kinase
PUFAs	polyunsaturated fatty acids
RNA	ribonucleic acid
ROS	reactive oxygen species
ROS/RNS	reactive oxygen/nitrogen species
SPHK1	sphingosine kinase 1
SOD	superoxide dismutase
SR-BI	scavenger receptor class I type B
SVR	sustained viral response
T2DM	type 2 diabetes mellitus
TC	total cholesterol
TCA	tricarboxylic acid
Tf	transferrin
Tf-Fe^3+^	monoferric transferrin
Tf-(Fe^3+^)_2_	differic transferrin
TfR	transferrin receptor
TfR1	transferrin receptor 1
TfR2	transferrin receptor 2
TG	triglycerides
TGF-β	transforming growth factor β
TIBC	total iron binding capacity
TIMPs	tecidular inhibitors of metalloproteinases
TNF-α	tumor necrosis factor alpha
TS	transferrin saturation
VLDL	very low-density lipoprotein
WHO	World Health Organization
γGT	gamma-glutamyl-transpeptidase
8-oxodG	8-oxo-deoxyguanosine
